# Supplementary material for: Refphase: Multi-sample phasing reveals haplotype-specific copy number heterogeneity
Source: PLoS Comput Biol. 2023 Oct 23;19(10):e1011379. doi: 10.1371/journal.pcbi.1011379 (PMC10621967; doi:10.1371/journal.pcbi.1011379)
Supplement: S1 Fig — The reference phasing algorithm phases individual segments across all samples but not segments with respect to each other. Therefore we employ a horizontal phasing algorithm which minimises the total number of copy-number breakpoints defined as the total Hamming distance between neighbouring segments (Eq 6). To this end we compare the Hamming distance between neighbouring segments with a flipped version of those segments (flipping a segment amounts to exchanging the haplotype assignment of that segment for all samples) to determine which segments need to be flipped. (PDF) [file pcbi.1011379.s001.pdf]

Supplementary Figures

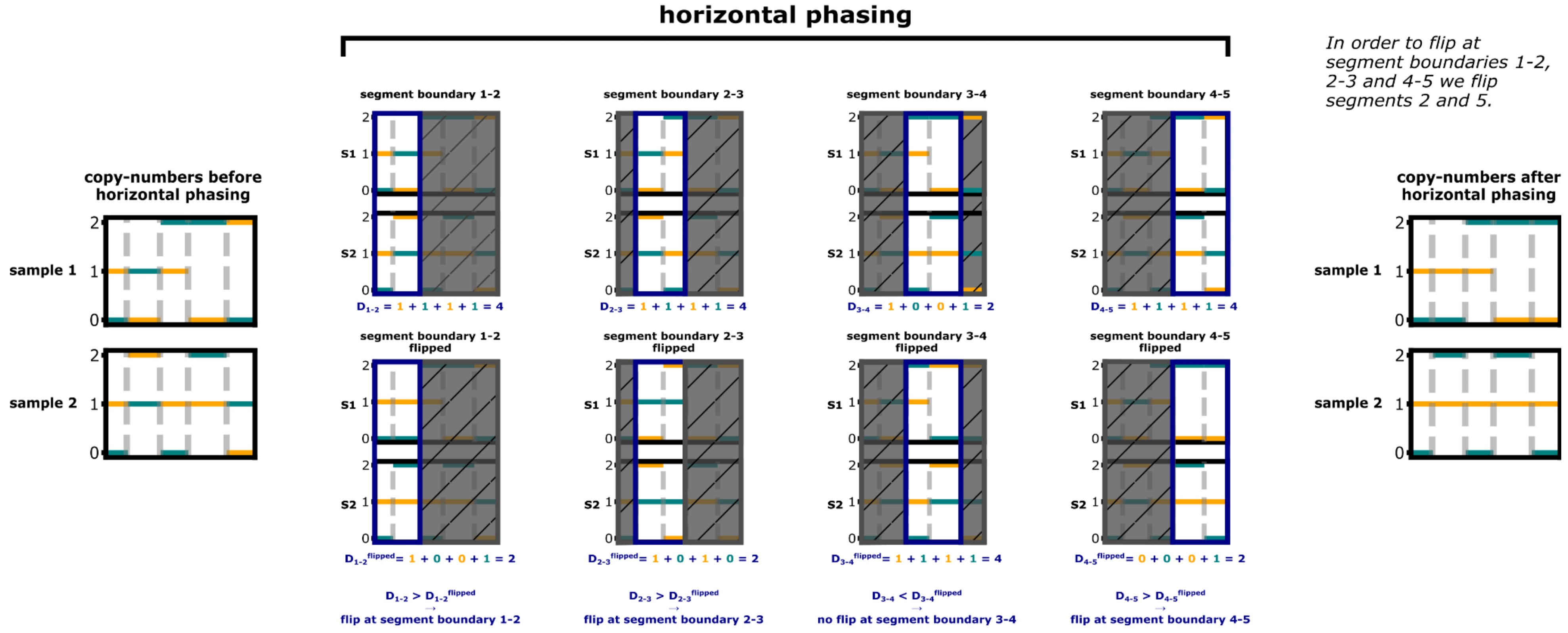

Supplementary Figure 1: Example for the horizontal phasing algorithm. The reference phasing algorithm phases individual segments across all samples but not segments with respect to each other. Therefore we employ a horizontal phasing algorithm which minimises the total number of copy-number breakpoints defined as the total Hamming distance between neighbouring segments (Eq. 6). To this end we compare the Hamming distance between neighbouring segments with a flipped version of those segments (flipping a segment amounts to exchanging the haplotype assignment of that segment for all samples) to determine which segments need to be flipped.
