# Supplementary material for: Refphase: Multi-sample phasing reveals haplotype-specific copy number heterogeneity
Source: PLoS Comput Biol. 2023 Oct 23;19(10):e1011379. doi: 10.1371/journal.pcbi.1011379 (PMC10621967; doi:10.1371/journal.pcbi.1011379)
Supplement: S2 Fig — The upper three panels show tracks for log read-depth ratio (LogR), B-Allele Frequency (BAF), re-estimated fractional copy number states, and somatic copy number aberration (SCNA) event calling at a sample level. The bottom panel (‘Summary’) gives a tumour-level summary of SCNA event clonality and detection of mirrored subclonal imbalance (MSAI), loss of heterozygosity (LOH) and parallel events. Events are called relative-to-ploidy using a mean logR threshold. (PDF) [file pcbi.1011379.s002.pdf]

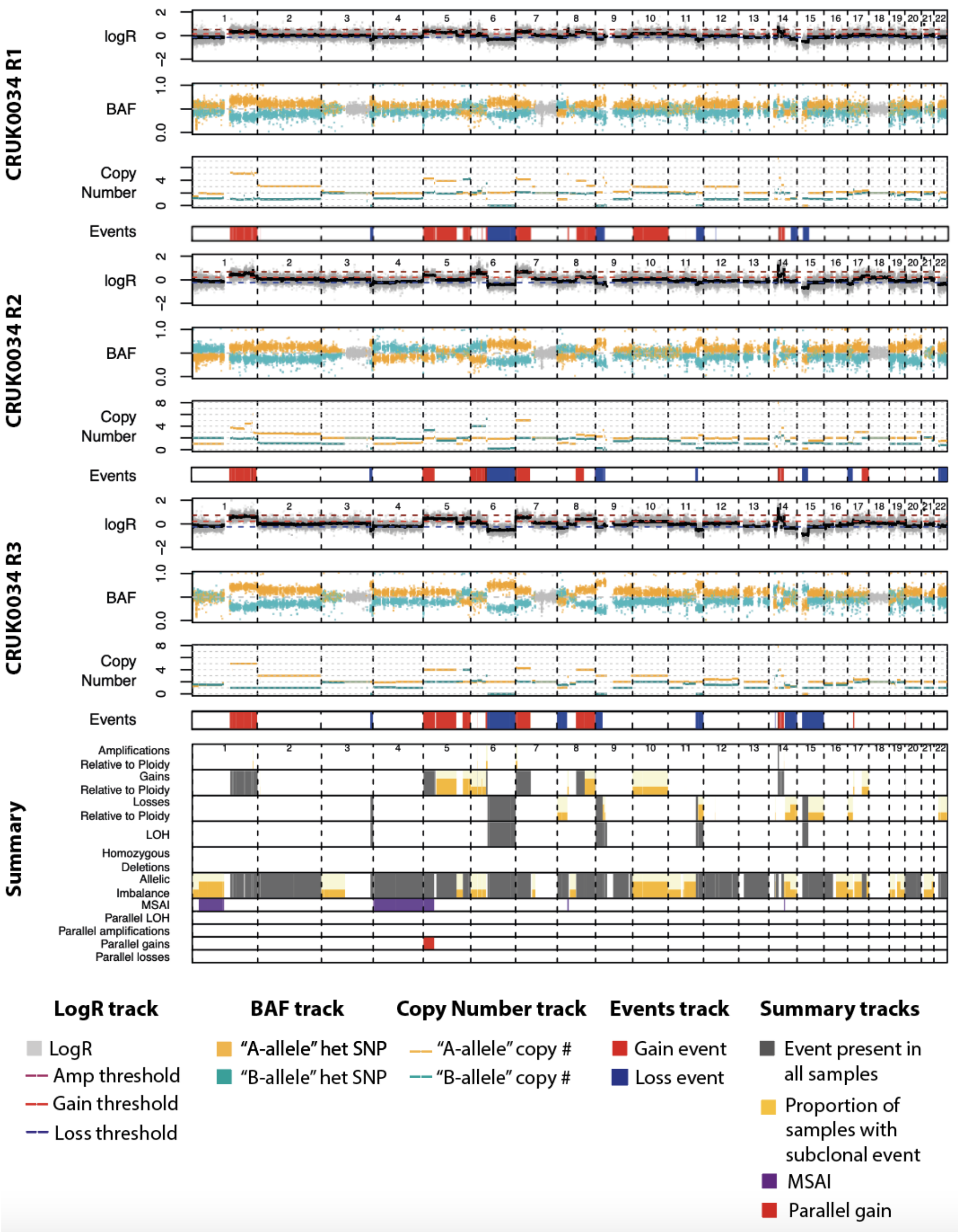

Supplementary Figure 2: Full Refphase across-genome plotting output for CRUK0034. The upper three panels show tracks for log read-depth ratio (LogR), B-Allele Frequency (BAF), re-estimated fractional copy number states, and somatic copy number aberration (SCNA) event calling at a sample level. The bottom panel ('Summary') gives a tumour-level summary of SCNA event clonality and detection of mirrored subclonal imbalance (MSAI), loss of heterozygosity (LOH) and parallel events. Events are called relative-to-ploidy using a mean logR threshold.
