# Supplementary material for: Refphase: Multi-sample phasing reveals haplotype-specific copy number heterogeneity
Source: PLoS Comput Biol. 2023 Oct 23;19(10):e1011379. doi: 10.1371/journal.pcbi.1011379 (PMC10621967; doi:10.1371/journal.pcbi.1011379)
Supplement: S3 Fig — The association between sample purity and proportion of genome with (a) allelic imbalance (LME coefficient = 0.10 (2.s.f.), LME ANOVA p = 0.03), (b) newly identified allelic imbalance (LME coefficient = -0.28 (2.s.f.), LME ANOVA p<0.0001), (c) allelic imbalance, as defined by Refphase and using original estimates from ASCAT (‘Original Total’), (d) MSAI (LME coefficient = 0.0045 (2.s.f.), LME ANOVA p = 0.5). (e) The association between mean tumour purity and proportion of genome with MSAI (LME coefficient = 0.070 (2.s.f.), LME ANOVA p = 0.07). Proportion of genome data is calculated using Refphase. Analyses are undertaken for the 336 tumour samples from 99 tumours in the pan-cancer cohort described in Fig 4 and summarised in S1 Table. Linear mixed effect (LME) coefficients and ANOVA p-values shown are adjusted for patient and study cohort (defined by tumour type and profiling platform) as random effects for sample-level analyses and study cohort only for tumour-level analysis, calculated using the nlme R package and maximum likelihood method. Best fit lines shown are derived using the LME model coefficient and intercept values. (PDF) [file pcbi.1011379.s003.pdf]

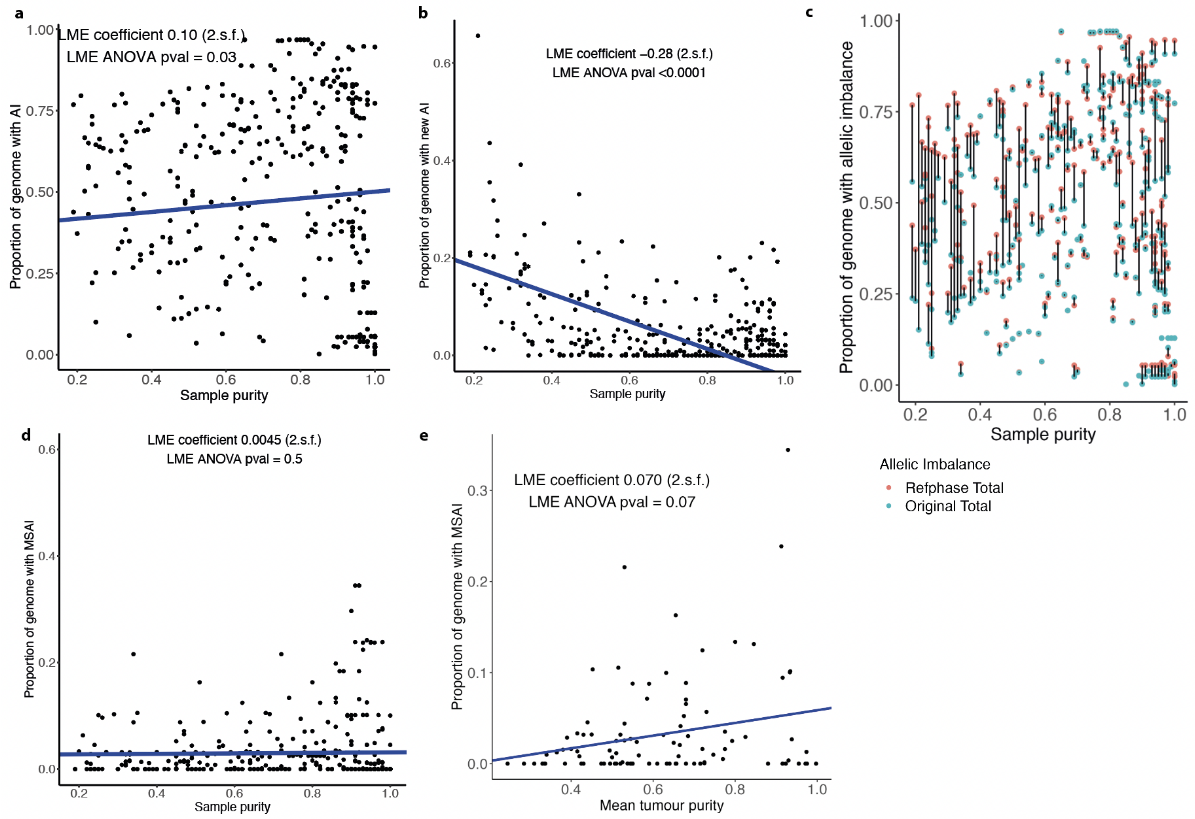

Supplementary Figure 3: Associations with sample purity. The association between sample purity and proportion of genome with **(a)** allelic imbalance (LME coefficient = 0.10 (2.s.f.), LME ANOVA p=0.03), **(b)** newly identified allelic imbalance (LME coefficient = -0.28 (2.s.f.), LME ANOVA p<0.0001), **(c)** allelic imbalance, as defined by Refphase and using original estimates from ASCAT ('Original Total'), **(d)** MSAI (LME coefficient = 0.0045 (2.s.f.), LME ANOVA p=0.5). (e) The association between mean tumour purity and proportion of genome with MSAI (LME coefficient = 0.070 (2.s.f.), LME ANOVA p=0.07). Proportion of genome data is calculated using Refphase. Analyses are undertaken for the 336 tumour samples from 99 tumours in the pan-cancer cohort described in Figure 4 and summarised in Supplementary Table S1. Linear mixed effect (LME) coefficients and ANOVA p-values shown are adjusted for patient and study cohort (defined by tumour type and profiling platform) as random effects for sample-level analyses and study cohort only for tumour-level analysis, calculated using the nlme R package and maximum likelihood method. Best fit lines shown are derived using the LME model coefficient and intercept values.
