# Supplementary material for: Refphase: Multi-sample phasing reveals haplotype-specific copy number heterogeneity
Source: PLoS Comput Biol. 2023 Oct 23;19(10):e1011379. doi: 10.1371/journal.pcbi.1011379 (PMC10621967; doi:10.1371/journal.pcbi.1011379)
Supplement: S4 Fig — a) An example of the effect of decreasing tumour purity on BAF band separation. b) The effect of varying copy number states on BAF band separation for a tumour of fixed purity, here 50%. c) Example of typical BAF and LogR profiles for a loss of heterozygosity (LOH) with total copy number loss (left) and copy-neutral LOH (CNLOH) (right). Relative-to-ploidy thresholds are indicated by dashed lines and are derived for a diploid 100% pure tumour using the formulae described in the Methods of this manuscript. Orange and blue points throughout plots represent the phased “A” haplotype and “B” haplotype respectively. (PDF) [file pcbi.1011379.s004.pdf]

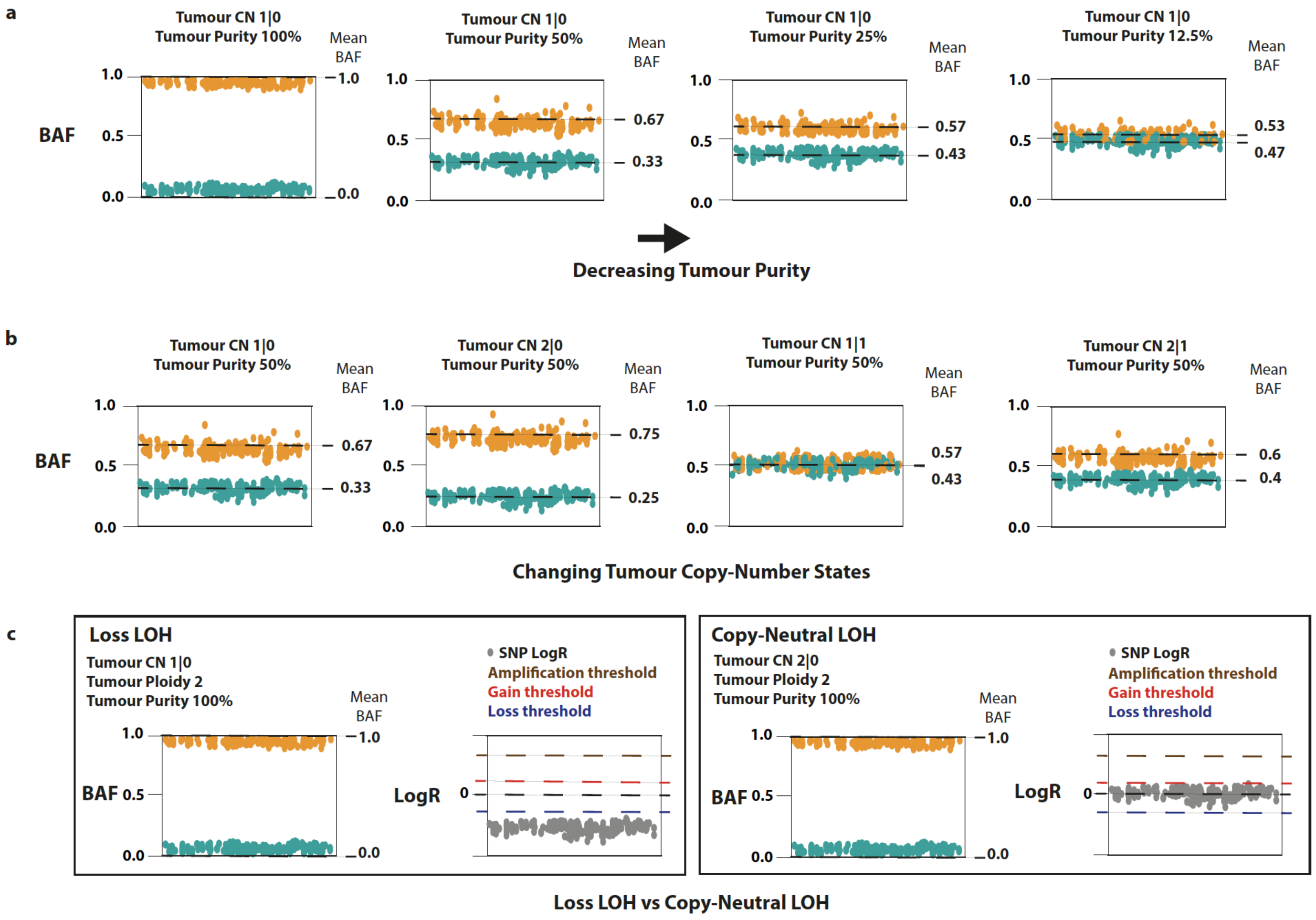


**Supplementary Figure 4: The effects of tumour purity and copy number states on B-Allele Frequency (BAF) and log read-depth ratio (LogR) profiles. a)** An example of the effect of decreasing tumour purity on BAF band separation. **b)** The effect of varying copy number states on BAF band separation for a tumour of fixed purity, here 50%. **c)** Example of typical BAF and LogR profiles for a loss of heterozygosity (LOH) with total copy number loss (left) and copy-neutral LOH (CNLOH) (right). Relative-to-ploidy thresholds are indicated by dashed lines and are derived for a diploid 100% pure tumour using the formulae described in the Methods of this manuscript. Orange and blue points throughout plots represent the phased “A” haplotype and “B” haplotype respectively.
