# Supplementary material for: Refphase: Multi-sample phasing reveals haplotype-specific copy number heterogeneity
Source: PLoS Comput Biol. 2023 Oct 23;19(10):e1011379. doi: 10.1371/journal.pcbi.1011379 (PMC10621967; doi:10.1371/journal.pcbi.1011379)
Supplement: S5 Fig — a) Proportion of the genome subject to subclonal SCNAs (ASCAT median = 0.28, Refphase median = 0.25, p = 3.2e-08). b) Proportion of aberrant genome subject to subclonal SCNAs, where aberrant genome is defined as the total length of genomic segments in which any SCNA event (clonal or subclonal) is called (ASCAT median = 0.48, Refphase median = 0.43, p = 8.5e-08). c) Proportion of genome subject to clonal loss of heterozygosity (LOH) (ASCAT median = 0.18, Refphase median = 0.22, p = 5.8e-13). d) Proportion of the genome subject to subclonal LOH (ASCAT median = 0.11, Refphase median = 0.07, p = 1.0e-13). All p-values shown are for paired Wilcoxon signed rank tests with continuity correction, across the n = 99 tumours described in Figs 4 and 5. SCNAs in (a) and (b) encompass relative-to-ploidy gains and losses, and LOH events. (PDF) [file pcbi.1011379.s005.pdf]

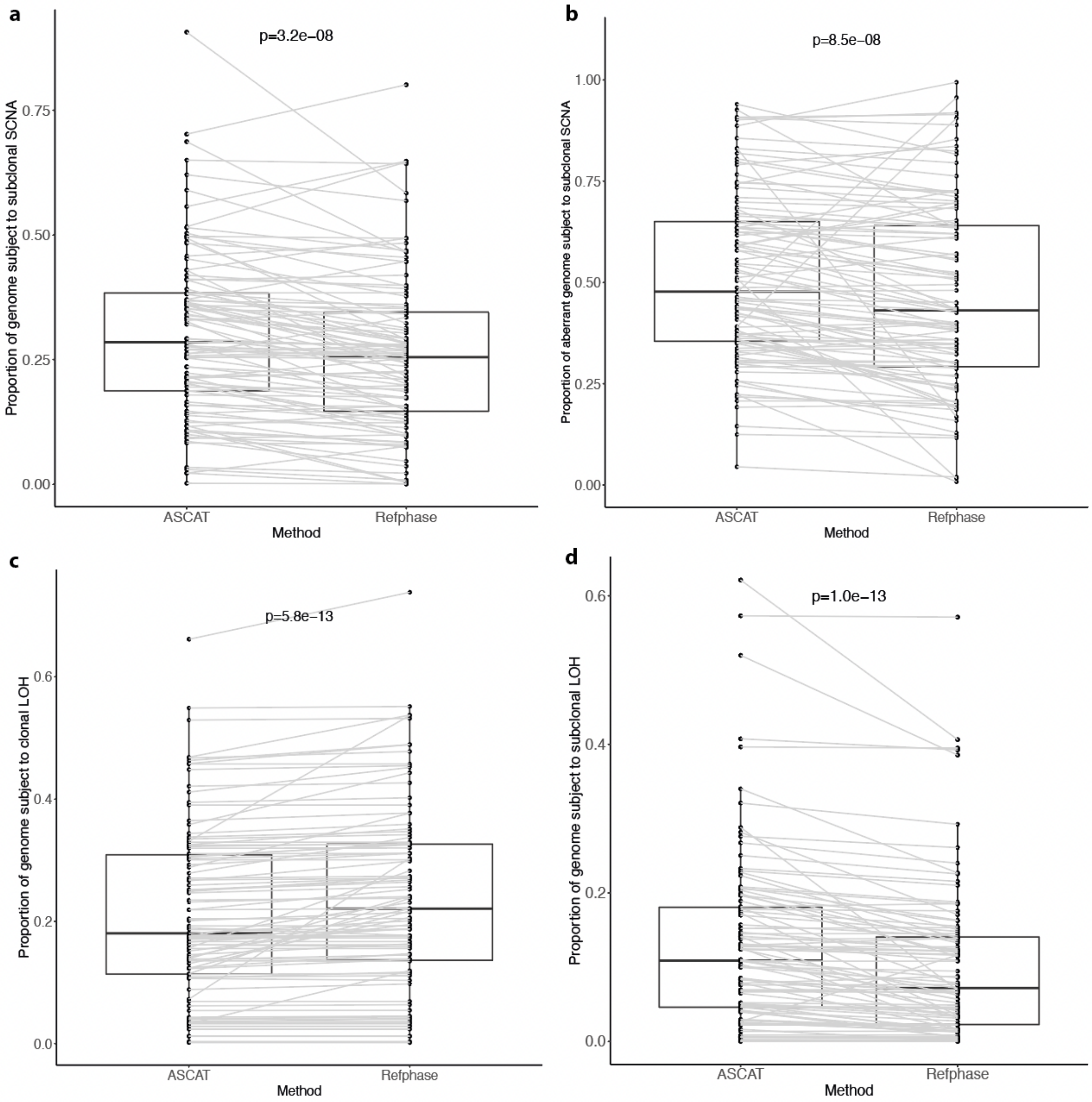

Supplementary Figure 5: Somatic Copy Number Aberration (SCNA) heterogeneity comparisons between ASCAT and Refphase. a) Proportion of the genome subject to subclonal SCNAs (ASCAT median = 0.28, Refphase median = 0.25, p=3.2e-08). **b)** Proportion of aberrant genome subject to subclonal SCNAs, where aberrant genome is defined as the total length of genomic segments in which any SCNA event (clonal or subclonal) is called (ASCAT median = 0.48, Refphase median = 0.43, p=8.5e-08). **c)** Proportion of genome subject to clonal loss of heterozygosity (LOH) (ASCAT median = 0.18, Refphase median = 0.22, p=5.8e-13). **d)** Proportion of the genome subject to subclonal LOH (ASCAT median = 0.11, Refphase median = 0.07, p=1.0e-13). All p-values shown are for paired Wilcoxon signed rank tests with continuity correction, across the n=99 tumours described in Figures 4 and 5. SCNAs in (a) and (b) encompass relative-to-ploidy gains and losses, and LOH events.
