# Supplementary material for: Refphase: Multi-sample phasing reveals haplotype-specific copy number heterogeneity
Source: PLoS Comput Biol. 2023 Oct 23;19(10):e1011379. doi: 10.1371/journal.pcbi.1011379 (PMC10621967; doi:10.1371/journal.pcbi.1011379)
Supplement: S6 Fig — Median proportions by Patient WGD Status (cWGD = 0.02, n = 54 tumours; sWGD = 0.07, n = 13 tumours; nWGD = 0, n = 32 tumours). Kruskal-Wallis p-value is shown (p = 2e-04). Data is shown for n = 99 tumours from the pan-cancer cohort showcased in Fig 5 for which MEDICC2 was used to infer WGD status. Proportion of genome data is assessed by Refphase. cWGD = clonal WGD; sWGD = subclonal WGD; nWGD = non-WGD. (PDF) [file pcbi.1011379.s006.pdf]

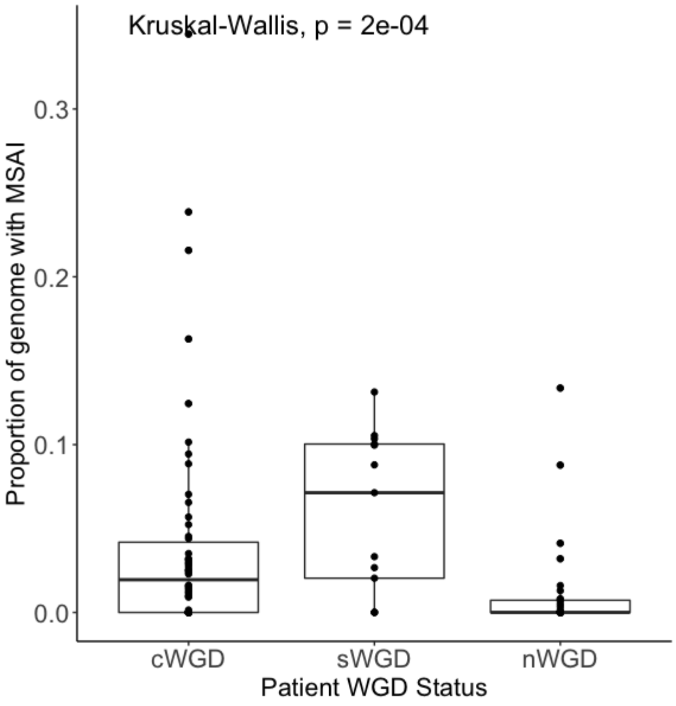

Supplementary Figure 6: Association between whole genome doubling (WGD) and mirrored subclonal allelic imbalance (MSAI). Median proportions by Patient WGD Status (cWGD = 0.02, n=54 tumours; sWGD = 0.07, n=13 tumours; nWGD = 0, n=32 tumours). Kruskal-Wallis p-value is shown (p=2e-04). Data is shown for n=99 tumours from the pan-cancer cohort showcased in Figure 5 for which MEDICC2 was used to infer WGD status. Proportion of genome data is assessed by Refphase. cWGD = clonal WGD; sWGD = subclonal WGD; nWGD = non-WGD.
