# Supplementary material for: Refphase: Multi-sample phasing reveals haplotype-specific copy number heterogeneity
Source: PLoS Comput Biol. 2023 Oct 23;19(10):e1011379. doi: 10.1371/journal.pcbi.1011379 (PMC10621967; doi:10.1371/journal.pcbi.1011379)
Supplement: S7 Fig — a) Examples of parallel gain events on chromosome arm 7p, observed between glands on different sides of the tumour. b) Example of a parallel gain event on chromosome 2, observed within glands on the same side of the tumour. Tumour IDs shown match those in the original publication [46]. (PDF) [file pcbi.1011379.s007.pdf]

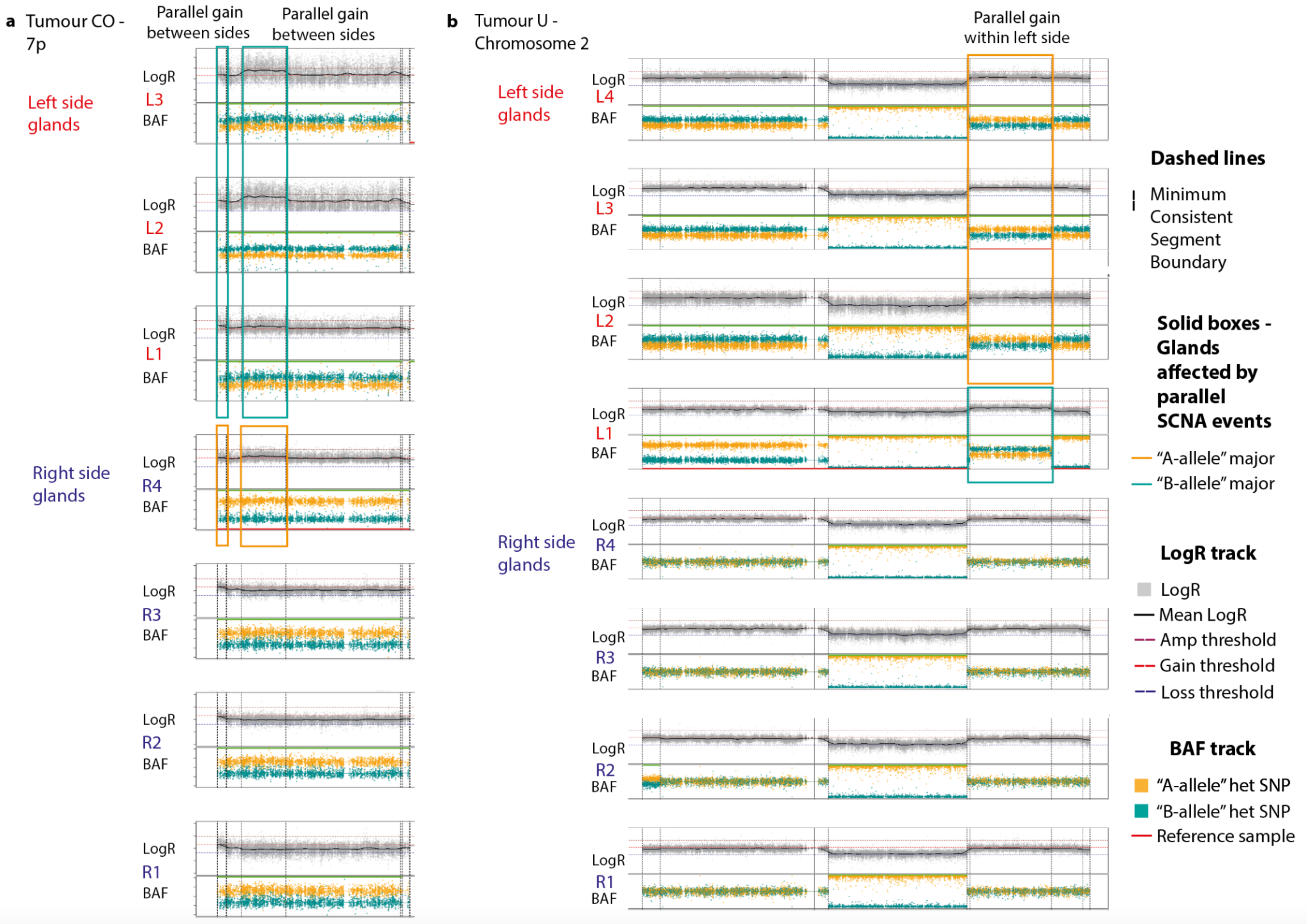

Supplementary Figure 7: Examples of parallel SCNA events in the Sottoriva *et al* [1] colorectal adenocarcinoma cohort. a) Examples of parallel gain events on chromosome arm 7p, observed between glands on different sides of the tumour. **b)** Example of a parallel gain event on chromosome 2, observed within glands on the same side of the tumour. Tumour IDs shown match those in the original publication [1].

References: 1. Sottoriva A, Kang H, Ma Z, Graham TA, Salomon MP, Zhao J, et al. A Big Bang model of human colorectal tumor growth. Nat Genet. 2015;47: 209–216.
