# Supplementary material for: Refphase: Multi-sample phasing reveals haplotype-specific copy number heterogeneity
Source: PLoS Comput Biol. 2023 Oct 23;19(10):e1011379. doi: 10.1371/journal.pcbi.1011379 (PMC10621967; doi:10.1371/journal.pcbi.1011379)
Supplement: S8 Fig — We ran MEDICC2 to infer the mutational events that occurred for each sample of the Brastianos and Sottoriva datasets, and used these events as input to a simple karyotype simulator. The simulation uniformly chooses a haplotype to apply the event to, and uniformly samples the starting locus for segmental events. The available phylogeny is then simulated, and the proportion of MSAI segments calculated (sum of MSAI segment lengths divided by sum of all segment lengths). Performing 350 simulations per sample, we generate the above violin plots in the same style as Fig 5C. The star data-point is then the sample’s actual/observed MSAI proportion. The corrected p-value of the one-sided right-tailed test is generated, and statistically significant observations are highlighted in blue. (PDF) [file pcbi.1011379.s008.pdf]

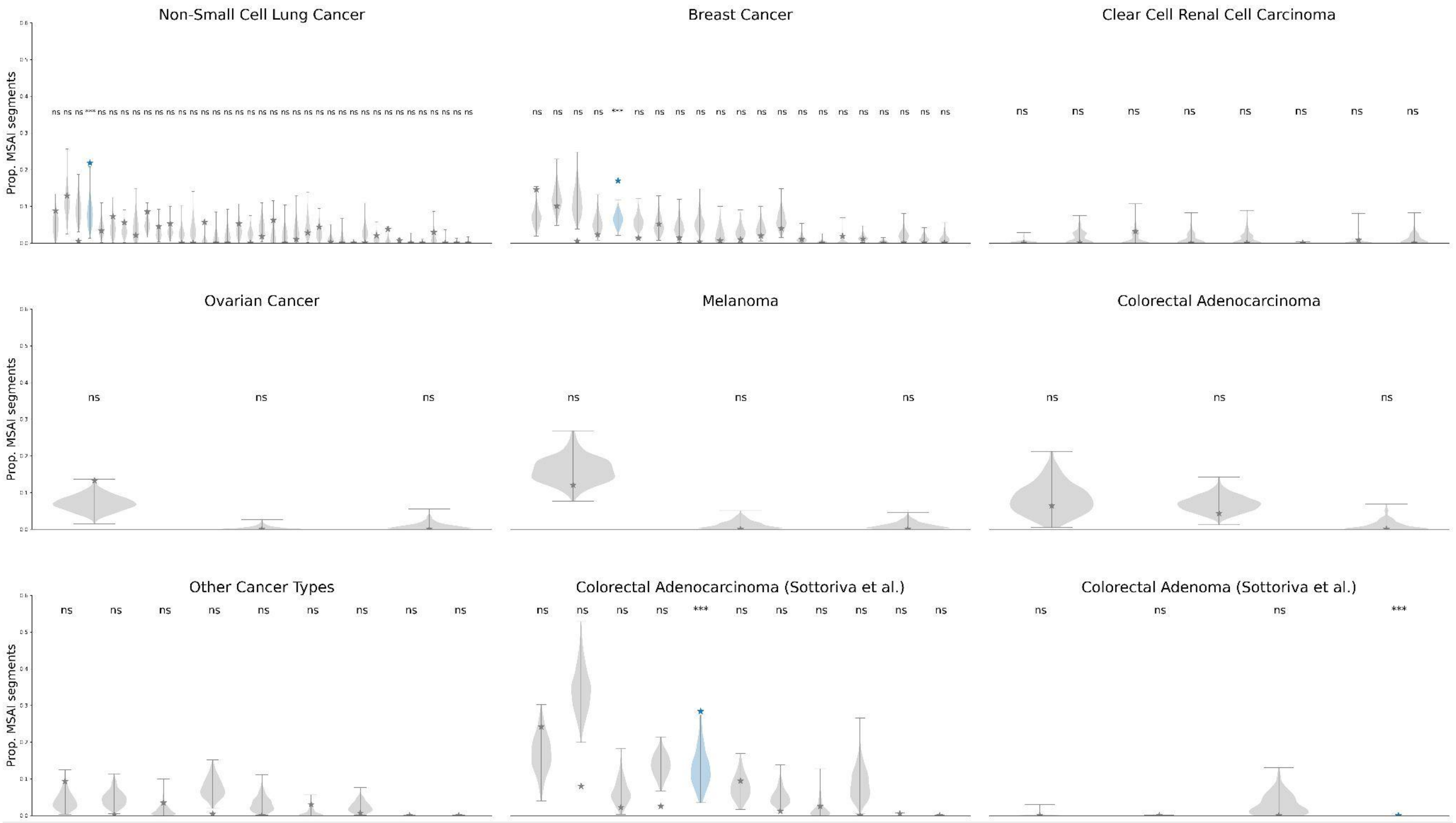

Supplementary Figure 8: Proportion of MSAI segments for the Brastianos and Sottoriva datasets versus simulated data. We ran MEDICC2 to infer the mutational events that occurred for each sample of the Brastianos and Sottoriva datasets, and used these events as input to a simple karyotype simulator. The simulation uniformly chooses a haplotype to apply the event to, and uniformly samples the starting locus for segmental events. The available phylogeny is then simulated, and the proportion of MSAI segments calculated (sum of MSAI segment lengths divided by sum of all segment lengths). Performing 350 simulations per sample, we generate the above violin plots in the same style as Fig. 5c. The star data-point is then the sample's actual/observed MSAI proportion. The corrected p-value of the one-sided right-tailed test is generated, and statistically significant observations are highlighted in blue.
