# Supplementary material for: Refphase: Multi-sample phasing reveals haplotype-specific copy number heterogeneity
Source: PLoS Comput Biol. 2023 Oct 23;19(10):e1011379. doi: 10.1371/journal.pcbi.1011379 (PMC10621967; doi:10.1371/journal.pcbi.1011379)
Supplement: S9 Fig — Association between proportion of aberrant genome with a) number of samples (LME coefficient = 0.017, LME ANOVA p = 0.1), and b) tumour purity difference—defined as the purity difference between the most and least pure sample within a tumour (LME coefficient = 0.28, LME ANOVA p = 0.04). Proportion of aberrant genome is defined as the proportion of the total length of genomic segments harbouring any relative-to-ploidy SCNA (gain or loss) or loss of heterozygosity (LOH) event which contains a subclonal SCNA or LOH event. Proportion of the aberrant genome is defined at the tumour level. Analyses are carried out for the pan-cancer cohort described in Figs 4 and 5 (n = 99 tumours). Linear mixed effect (LME) ANOVA p-values and LME coefficients calculated using the nlme R package are shown, with analyses adjusted for study cohort (defined by histology and sequencing platform), indicated by the colour legend. Whole-exome sequencing (WES) data is taken from the Brastianos et al. study [47]; SNP array data is taken from the Sottoriva et al. study [46]. Best fit lines shown have LME slope and intercept values. (PDF) [file pcbi.1011379.s009.pdf]

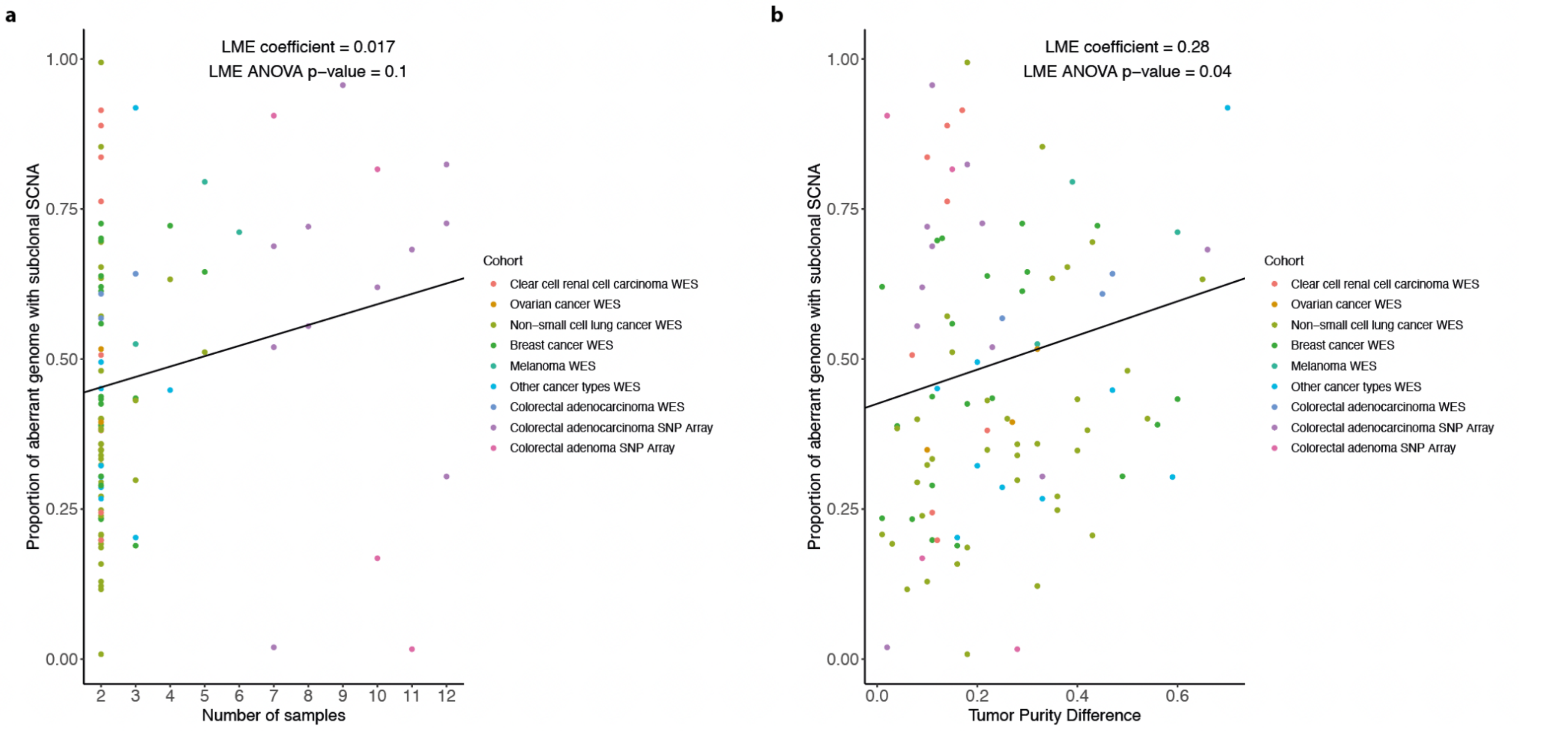

Supplementary Figure 9: The relationship between SCNA intra-tumour heterogeneity and clinical variables. Association between proportion of aberrant genome with **a)** number of samples (LME coefficient = 0.017, LME ANOVA p=0.1), and **b)** tumour purity difference - defined as the purity difference between the most and least pure sample within a tumour (LME coefficient = 0.28, LME ANOVA p=0.04). Proportion of aberrant genome is defined as the proportion of the total length of genomic segments harbouring any relative-to-ploidy SCNA (gain or loss) or loss of heterozygosity (LOH) event which contains a subclonal SCNA or LOH event. Proportion of the aberrant genome is defined at the tumour level. Analyses are carried out for the pan-cancer cohort described in Figures 4 and 5 (n=99 tumours). Linear mixed effect (LME) ANOVA p-values and LME coefficients calculated using the nlme R package are shown, with analyses adjusted for study cohort (defined by histology and sequencing platform), indicated by the colour legend. Whole-exome sequencing (WES) data is taken from the Brastianos *et al.* study [1]; SNP array data is taken from the Sottoriva *et al.* study [2]. Best fit lines shown have LME slope and intercept values.

References:

1. Brastianos PK, Carter SL, Santagata S, Cahill DP, Taylor-Weiner A, Jones RT, et al. Genomic Characterization of Brain Metastases Reveals Branched Evolution and Potential Therapeutic Targets. Cancer Discov. 2015;5: 1164–1177.
2. Sottoriva A, Kang H, Ma Z, Graham TA, Salomon MP, Zhao J, et al. A Big Bang model of human colorectal tumor growth. Nat Genet. 2015;47: 209–216.
