# Supplementary material for: Refphase: Multi-sample phasing reveals haplotype-specific copy number heterogeneity
Source: PLoS Comput Biol. 2023 Oct 23;19(10):e1011379. doi: 10.1371/journal.pcbi.1011379 (PMC10621967; doi:10.1371/journal.pcbi.1011379)
Supplement: S10 Fig — Barcharts for each sample in the Gundem et al. [53] WGS cohort showing concordance and discordance between Refphase derived integer copy number and dominant subclone number from Battenberg and HATCHet showing: a) total copy number across all segments b) total copy number across all segments without within-sample subclonal copy number called by both Battenberg and HATCHet c) total copy number across all segments that demonstrate within-sample subclonal copy number called by both Battenberg and HATCHet.d) Major copy number across all segments e) Major copy number across all segments without within-sample subclonal copy number called by both Battenberg and HATCHet f) Major copy number across all segments that demonstrate within-sample subclonal copy number called by both Battenberg and HATCHet. g) Minor copy number across all segments h) Minor copy number across all segments without within-sample subclonal copy number called by both Battenberg and HATCHet i) Minor copy number across all segments that demonstrate within-sample subclonal copy number called by both Battenberg and HATCHet. (PDF) [file pcbi.1011379.s010.pdf]

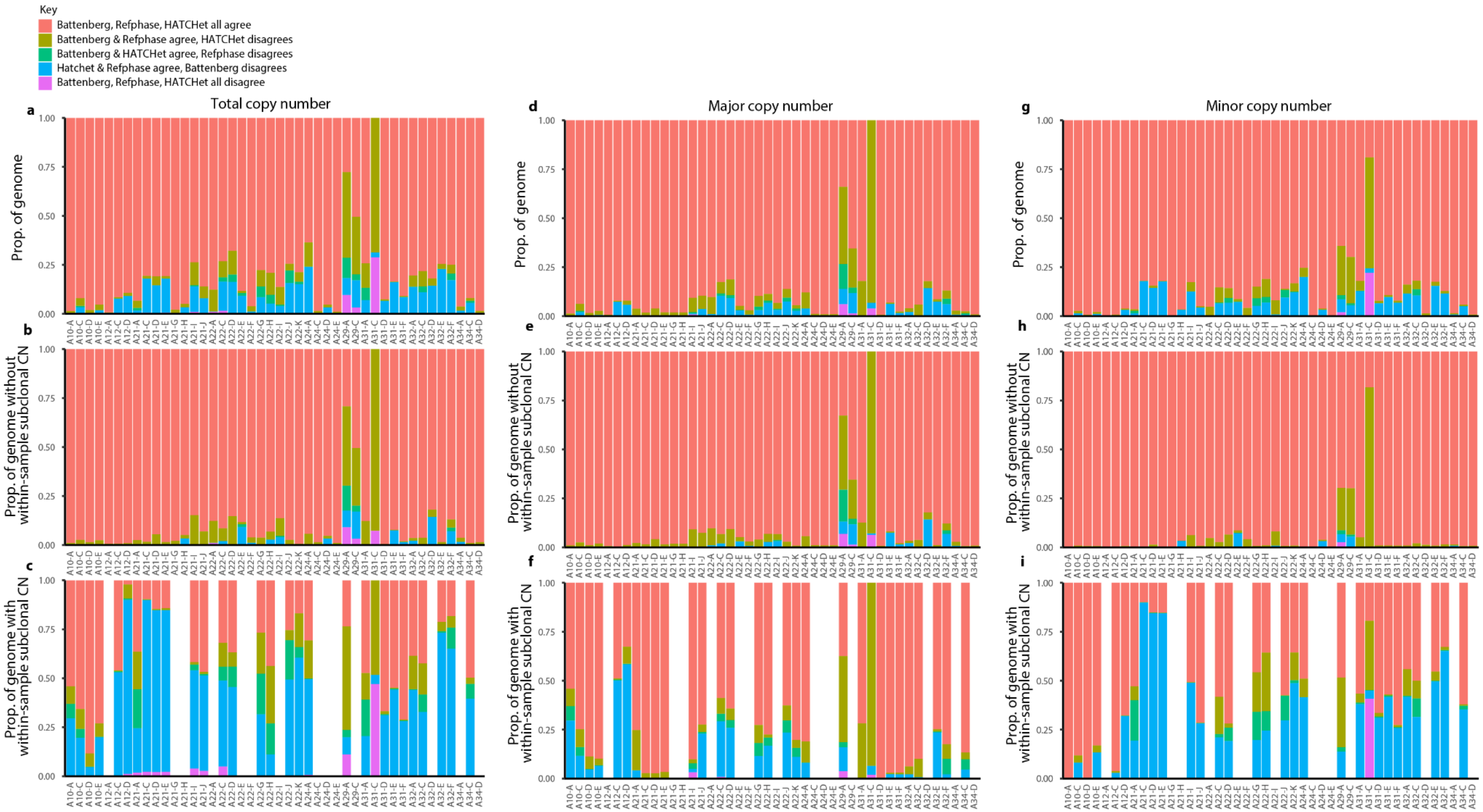

Supplementary Figure 10: Refphase, Battenberg, and HATCHet comparisons. Barcharts for each sample in the Gundem et al. WGS cohort showing concordance and discordance between Refphase derived integer copy number and dominant subclone number from Battenberg and HATCHet showing: **a)** total copy number across all segments **b)** total copy number across all segments without within-sample subclonal copy number called by both Battenberg and HATCHet **c)** total copy number across all segments that demonstrate within-sample subclonal copy number called by both Battenberg and HATCHet.**d)** Major copy number across all segments **e)** Major copy number across all segments without within-sample subclonal copy number called by both Battenberg and HATCHet **f)** Major copy number across all segments that demonstrate within-sample subclonal copy number called by both Battenberg and HATCHet. **g)** Minor copy number across all segments **h)** Minor copy number across all segments without within-sample subclonal copy number called by both Battenberg and HATCHet **i)** Minor copy number across all segments that demonstrate within-sample subclonal copy number called by both Battenberg and HATCHet.
